# Incidental Presacral Myelolipoma Resembling the Liposarcoma: A Case Report and Literature Review

**DOI:** 10.1155/2016/6510930

**Published:** 2016-12-28

**Authors:** Naoto Tokuyama, Hisashi Takeuchi, Isao Kuroda, Teiichiro Aoyagi

**Affiliations:** Department of Urology, Tokyo Medical University Ibaraki Medical Center, Ibaraki, Japan

## Abstract

Presacral myelolipomas are rare, benign, asymptomatic tumors composed of mature adipose tissue and hematopoietic elements, but fewer than 50 cases have been reported in the literature. They are usually discovered incidentally during imaging studies and are often misdiagnosed as liposarcoma, which have a malignant nature, because the imaging findings of myelolipoma can be similar to those of liposarcoma. It is challenging to distinguish presacral myelolipomas from other presacral fat-containing tumors without performing a histological examination. We should consider the possibility of a malignant tumor, and imaging-guided biopsy carries a risk of tumor spread along the biopsy tract. Therefore, surgical management might sometimes be required; however, it is not necessary in all cases. We present an incidentally detected case of presacral myelolipoma that was difficult to differentiate from other malignant tumors in a 71-year-old male.

## 1. Introduction

Myelolipomas are rare, benign tumors composed of mature adipose tissue and hematopoietic elements. They do not usually produce any symptoms and are often discovered incidentally on imaging studies. Myelolipoma almost always occurs in the adrenal glands, but some extra-adrenal cases have been reported in the literature [1–4]. Presacral myelolipoma accounts for most of these cases. It is very difficult to differentiate presacral myelolipomas from other masses, including liposarcomas, teratomas, extramedullary hematopoiesis, and neurogenic tumors at presacral locations [5–8]. Among these lesions, liposarcoma is the most common fat-containing tumor with a malignant nature. In cases involving presacral mass lesions, surgical management should be considered unless the patient is diagnosed with a distinctly benign tumor, such as lipoma [6]. We reported a case of presacral myelolipoma that was difficult to differentiate from other malignant tumors in an elderly male.

## 2. Case Presentation

A 71-year-old male underwent abdominal computed tomography (CT) as part of the first follow-up of an intraductal papillary mucinous neoplasm of the pancreas. CT demonstrated a presacral mass, which was suspected to be a liposarcoma. The patient was referred to our hospital for further examination and treatment.

The patient had no remarkable symptoms. His medical history included alcoholic liver disease. On physical examination, his abdomen was soft, and no palpable masses were detected. There were no significant signs of sacral root involvement, such as defecation or urination disorders, and there were no abnormalities in the patient's laboratory data.

**Figure 1:**
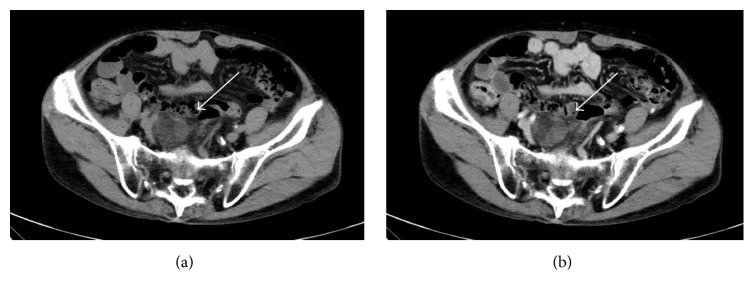
Enhanced CT scan showed a heterogenous round mass in the presacral space (diameter: 43 mm) that contained low-density areas ((a) plane, (b) arterial phase).

**Figure 2:**
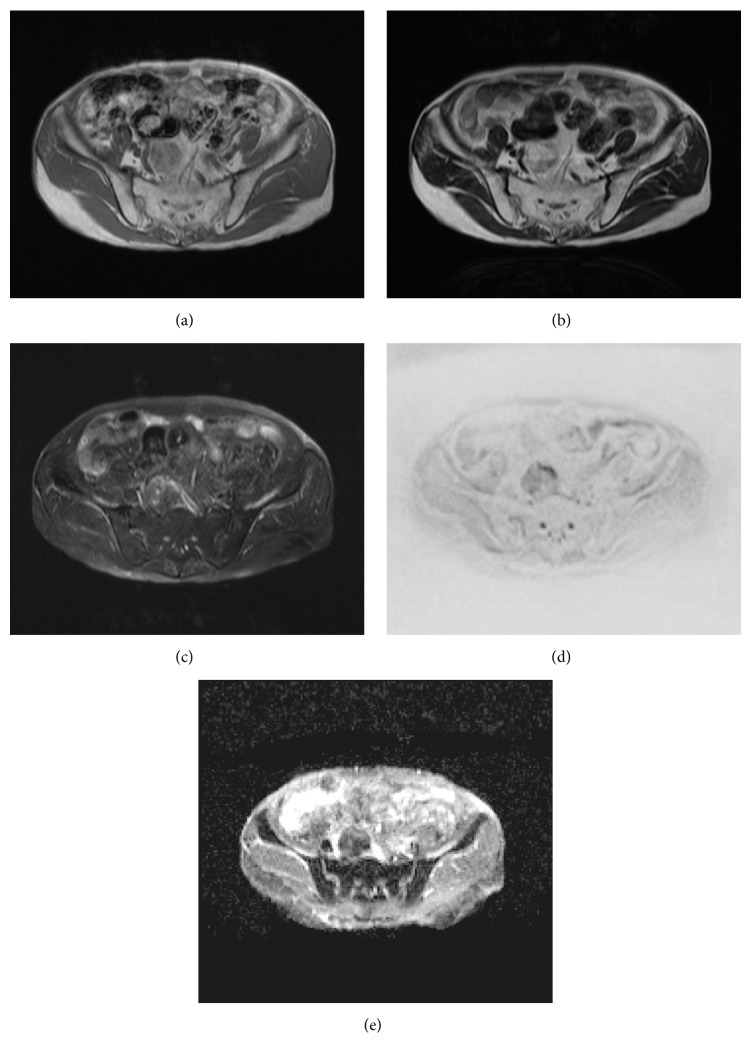
MR images showed a round heterogenous tumor in the presacral space ((a) T1-weighted, (b) T2-weighted, (c) fatty-saturated T2-weighted, and (d) diffusion-weighted image (*b* = 800) and (e) ADC map).

Contrast-enhanced CT was performed, which revealed a heterogenous mass in the presacral space (diameter: 43 mm) that contained low-density areas suggestive of fatty components and some slight enhanced areas suggestive of soft tissue islands inside the mass (Figure 1). Subsequent pelvic magnetic resonance imaging (MRI) showed a 43 mm mass in the anterior aspect of the sacrum (Figure 2). The tumor exhibited heterogeneous high intensity on T1- and T2-weighted images. T1-weighted chemical shift out-of-phase imaging demonstrated signal loss within the mass. No significant restricted diffusion was detected on diffusion-weighted images and ADC map. The tumor was considered to be composed of fatty tissue. There was no invasion to the adjacent structures, such as the sacral cortex, nerve roots, or pelvic lymph nodes.

Based on the patient's imaging findings, surgical resection was performed under a provisional diagnosis of liposarcoma. No preoperative biopsy was carried out due to a concern about potential biopsy tract malignant seeding.

**Figure 3:**
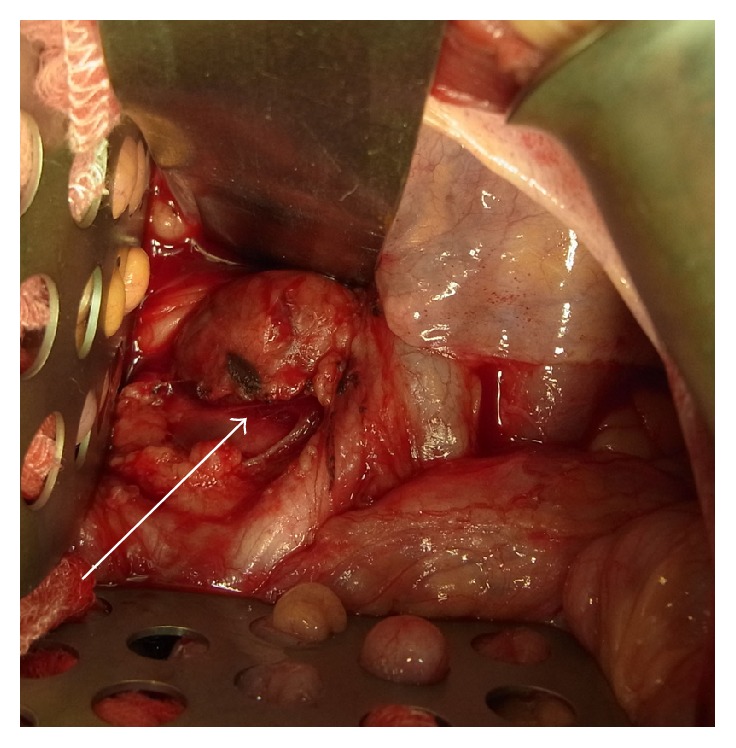
A tumor located in the anterior of the sacrum and consisting of soft, yellowish tissue. The mass was poorly defined and strongly adherent to the sacrum and the common iliac vein.

**Figure 4:**
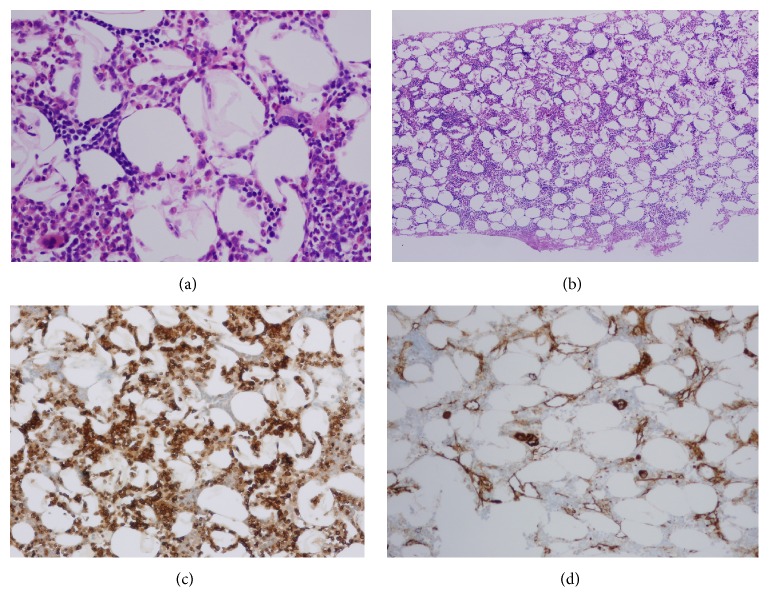
Histopathology of the tumor shows mature adipose tissue mixed with hematopoietic tissue, which included erythrocytes, myelocytes, and megakaryocytes. Immunohistochemical staining confirmed the presence of hematopoietic elements consisting of myeloperoxidase-positive myeloid elements and factor VIII-positive megakaryocytes (HE stain (a) ×400, (b) ×40, (c) myeloperoxidase-positive myeloid elements, and (d) factor VIII-positive megakaryocytes).

The surgery was performed under general anesthesia, and the abdomen was entered via a lower midline incision. The tumor was located between the anterior of the sacrum and the visceral pelvic fascia and consisted of soft, yellowish tissue. The mass was poorly defined and strongly adherent to the sacrum and the common iliac vein (Figure 3). Complete surgical resection of the entire tumor was considered to be difficult so we changed to open biopsy. The patient recovered well from the surgery. A histopathological examination showed mature adipose tissue mixed with hematopoietic tissue, which included erythrocytes, myelocytes, and megakaryocytes. Immunohistochemical staining confirmed the presence of hematopoietic elements consisting of myeloperoxidase-positive myeloid elements and factor VIII-positive megakaryocytes (Figure 4). There was no evidence of malignancy. These findings were consistent with presacral myelolipoma.

## 3. Discussion

Myelolipomas were first reported in 1905 by Gierke [9].

Myelolipomas are benign, most frequently occur in the adrenal glands, and have a low growth rate. After the adrenal glands, myelolipomas most commonly occur as presacral lesions. Approximately 15% of myelolipomas arise as extra-adrenal lesions [10]. Singla et al. reported a summary of 37 cases of extra-adrenal myelolipomas. The mean (and standard deviation) age of the patients was 65.2 ± 11.2 years, and the male to female ratio was 0.54 (male : female = 13 : 24). The reported extra-adrenal myelolipomas occurred at the following sites: the presacral space (*n* = 15, 40.5%), the retroperitoneum (*n* = 8, 21.6%), the thoracic cavity (*n* = 5, 13.5%), the pelvic cavity (*n* = 3, 8.1%), the kidneys (*n* = 2, 5.4%), the stomach (*n* = 1, 2.7%), the liver (*n* = 1, 2.7%), the bladder (*n* = 1, 2.7%), and multiple lesions (*n* = 1, 2.7%) [3]. Fewer than 50 cases of presacral myelolipoma have been described in the literature.

Presacral myelolipomas are often indolent, produce no symptoms, and are usually found incidentally during the follow-up of another disease or the examination of vague abdominal symptoms. In cases in which the tumors grow gradually and are large in diameter, some symptoms can appear, such as distention, anorexia, and abdominal pain. There is a possibility of tumor rupture and bleeding.

The detection of mature adipose tissue mixed with hematopoietic elements that include erythrocytes, myelocytes, and megakaryocytes during histopathological examinations is necessary in order to obtain a definitive diagnosis. It is generally reported that myelolipoma has a good prognosis in the absence of metastasis.

The imaging features of myelolipoma are based on the fact that such tumors consist of fatty components. On ultrasound sonography, myelolipomas display heterogeneous echogenicity and appear as well-circumscribed masses. They do not demonstrate blood flow signals on Doppler examination. On CT imaging, they exhibit low-density fatty components mixed with areas of soft tissue density representing hematopoietic tissue [11]. Myelolipomas do not invade adjacent structures. However, the adjacent structures can be compressed depending on the size of the tumor. The hematopoietic tissue components of myelolipomas can be enhanced on contrast-enhanced CT. Regarding MRI imaging, the fatty components of myelolipomas exhibit high intensity on T1- and T2-weighted images, whereas they demonstrate signal loss on fat-suppressed sequences. The hematopoietic elements display low-to-intermediate intensity on T1-weighted images and intermediate-to-high intensity on T2-weighted images [12–14]. The presence of intratumoral hemorrhaging might alter the imaging findings of myelolipoma [15]. Sometimes, the imaging appearance of myelolipoma can be similar to that of liposarcoma. As a result, myelolipomas are often misdiagnosed as liposarcoma during imaging studies [6, 16]. In our case, both CT and MRI are very good identifying lesions characterized by predominant adipose tissue. We initially diagnosed the mass as a liposarcoma. Differentiating presacral myelolipomas from other fat-containing presacral tumors (e.g., liposarcomas, teratomas, or extramedullary hematopoiesis) can be challenging [4–7]. Other differential diagnoses for a presacral mass include neurogenic tumor, which is not a fat-containing tumor. Liposarcoma is the most common retroperitoneal fat-containing tumor. We must consider the possibility of liposarcoma when we find a retroperitoneal fat-containing tumor because liposarcomas have a malignant nature and exhibit more aggressive progression than myelolipoma. Both presacral myelolipoma and liposarcoma have fatty components mixed with soft tissue elements and can be difficult to distinguish from one another on imaging studies. Thus, a histopathological examination is necessary to obtain a correct diagnosis. Several studies have reported that CT- or US-guided fine needle biopsy examinations are useful for acquiring complementary diagnostic information [17–20]; however, there is a risk of tumor spread along the biopsy tract and sampling error. Clinician should be aware of the risks of malignant seeding, bleeding, and sampling errors during percutaneous or transrectal biopsy procedures [3, 4]. If a tumor is suspected of being malignant or symptomatic or cannot be diagnosed at the time of biopsy, surgical resection might be necessary [21]. Even if the tumor is a liposarcoma, which has a malignant nature, surgical resection with a sufficient margin can be appropriate.

In this case, we initially intended to treat the patient via surgical resection; however, we abandoned the total resection because the tumor was found to be ill-defined and adherent to the sacrum and common iliac vein during the intraoperative examination. Similar to our case, it has been reported that extra-adrenal myelolipomas are difficult to separate from adjacent structures intraoperatively. Liu et al. proposed that intraoperative frozen sections play an important role in determining the extent of surgery. If it is expected that total resection will be challenging, the acquisition of intraoperative frozen sections should be considered to avoid complications [22].

In conclusion, presacral myelolipomas are rare and benign and have a good prognosis. It is challenging to differentiate myelolipomas from other presacral tumors that have a malignant nature, such as liposarcoma, without performing a histological examination. Many surgeons are concerned about the possibility of seeding malignancy and sampling error. Thus, they often tend to choose surgical management for diagnosis and treatment; however, it is not necessary in all cases. Image-guided biopsy sometimes might be required for diagnostic purposes and could help to prevent unnecessary surgical interventions; however we also should consider a risk of tumor spread along the biopsy tract, bleeding, and sampling error during biopsy procedure. We reported a case of presacral myelolipoma that was discovered incidentally and reviewed the relevant literature.
